# A Novel Population of *Fusarium* *fujikuroi* Isolated from Southeastern U.S. Winegrapes Reveals the Need to Re-Evaluate the Species’ Fumonisin Production

**DOI:** 10.3390/toxins8090254

**Published:** 2016-08-31

**Authors:** Stephanie L. Bolton, Phillip M. Brannen, Anthony E. Glenn

**Affiliations:** 1 Department of Plant Pathology, The University of Georgia, Athens, GA 30602, USA; stephbolton@gmail.com (S.L.B.); pbrannen@uga.edu (P.M.B.); 2 U.S. National Poultry Research Center, R.B. Russell Research Center, USDA-ARS, Toxicology and Mycotoxin Research Unit, Athens, GA 30605, USA

**Keywords:** fumonisin, *Fusarium*, *Fusarium fujikuroi*, grapes, ochratoxin A, *Vitis vinifera*, *Fusarium proliferatum*

## Abstract

Mycotoxins pose a challenge to a safe food supply worldwide, and their threat is expected to worsen with our changing climate. The need for diligence is exemplified by the discovery of fumonisin B2 in wine, which joins ochratoxin A as a mycotoxin of concern in the grape-wine chain. To elucidate the mycotoxin risk in southeastern American wine, grape samples were collected from vineyards during harvest in 2013 and potentially mycotoxigenic fungi (*Fusarium* and *Aspergillus*) were isolated from the samples. Numerous *Fusarium* isolates were recovered and identified to the species level by comparison of translation elongation factor 1-α gene sequences to verified strains. *Fusarium* *fujikuroi* was the most abundant species recovered (239 isolates), followed by *F. proliferatum* (52), *F. incarnatum*-*equiseti* (14), *F. oxysporum* (7), *F. concentricum* (1), and *F. solani* (1). In vitro assays quantified fumonisin production for representative isolates via liquid chromatography-tandem mass spectrometry. Surprisingly, nearly all *F. fujikuroi* isolates produced fumonisins B1, B2, and B3 at levels comparable to both the *F. proliferatum* isolates and the positive control, *Fusarium* *verticillioides*. Such capacity for fumonisin production refutes the generally accepted notion that *F.* *fujikuroi* produces undetectable or low levels of fumonisins and provides evidence to reconsider this species as a mycotoxigenic threat to economically significant crops.

## 1. Introduction

The possibility exists that mycotoxins, toxins produced by fungi having adverse effects on humans and animals, can survive the winemaking process to contaminate bottled wine even after the fungi themselves are eliminated from the must. The mycotoxin ochratoxin A (OTA) was discovered in 1965 by van der Merwe et al. [1], and the mycotoxins fumonisin B1 (FB1) and fumonisin B2 (FB2) were identified in 1988 [2]. In 1996, Zimmerli and Dick discovered OTA in wine [3] and more recently, in 2010 FB2 was revealed as a wine contaminant [4,5]. OTA, a mycotoxin produced by black-spored *Aspergillus* species such as *Aspergillus carbonarius* and *Aspergillus niger* [6,7,8], and present in cereals, wine, and coffee, has either been shown to be or is potentially nephrotoxic, immunosuppressive, teratogenic, genotoxic, embryotoxic, and cytotoxic [9]. OTA is also a possible human carcinogen in Group 2B, with fellow members dichlorodiphenyltrichloroethane (DDT), lead, and chloroform [10]. The source of FB2 in wine has been attributed to *A. niger* [11,12]. This is in contrast to FB1 in maize, which is the most common fumonisin in that commodity and is produced mainly by *Fusarium verticillioides* [13]. FB1 is noted as neurotoxic, hepatotoxic, and nephrotoxic in animals, and joins OTA as a possible Group 2B carcinogen [14].

Winegrapes are well established as a host for potentially mycotoxigenic black-spored aspergilli [15], but only a few reports have noted *Fusarium* species on winegrapes. One Slovakian survey revealed the presence of *Fusarium proliferatum* on grapes [16]. A second Slovakian survey isolated *F. proliferatum* from both surface-sterilized and non-sterilized grape berries, indicating that the fungus is capable of being present not only on the exterior of the grapes, but also within the berries [17]. A survey of Japanese wines found minor contamination (less than their limit of quantification, 1 ppb) with fumonisin B1 in a few samples [18] and later attributed this existence to *F. proliferatum* [19].

Only a few *Fusarium* species can produce the B series of fumonisins in significant concentrations, with *F. verticillioides* and *F. proliferatum* being the most notable. Despite having a fumonisin biosynthetic gene cluster (FUM) nearly identical to that of *F. verticillioides* and *F. proliferatum* [20], *Fusarium fujikuroi* is widely noted in the literature to produce low amounts, if any, of B-type fumonisins except in rare cases [21,22,23,24,25]. Interestingly, Suga et al. [26] found a single nucleotide polymorphism (SNP) in the translation elongation factor 1-α (TEF) gene of *F. fujikuroi* that correlated with minor fumonisin production in all Japanese isolates studied. This species is best known as a pathogen of rice contributing to Bakanae disease, or the “foolish seedling disease,” caused by the gibberellin secondary metabolites produced by the fungus [27].

The southeastern American states currently producing commercially available red *Vitis vinifera* grapes are Alabama, Georgia, North Carolina, Tennessee, Kentucky, and Virginia. Popular grape varieties are Cabernet franc, Cabernet Sauvignon, and Merlot, although other varieties such as Sangiovese, Malbec, and Tannat are also grown. The Southeast represents a challenging environment for winegrapes, due to both disease and weather. Pierce’s disease, vectored by the glassy-winged sharpshooter insect, is widespread, and a strict fungicide regimen is generally necessary to mitigate the effects of mildews and rots [28]. Weather challenges include late-season rains, hail, devastating spring frosts, and even hurricanes. Plant pathogens, insects, and poor weather may all contribute to a significant level of micro-punctured or wounded berries in the vineyard, which allow for easier access to berry nutrients by opportunistic fungal pathogens, such as black-spored *Aspergillus* and *Fusarium* spp. [29]. In fact, *A. carbonarius* produces OTA more efficiently on damaged berries than on intact ones [30]. Since winegrapes in the U.S. have the potential to be contaminated by mycotoxins, especially in the southeastern region where the climate is conducive to the growth of *Aspergillus* and *Fusarium* species, the presence of mycotoxigenic fungi in the vineyards was evaluated in the interest of public health safety and to fill a knowledge gap since vineyards within this region have not been extensively surveyed for these fungi. Thus, the objectives of this work were to isolate potentially mycotoxigenic fungi from southeastern U.S. red *V. vinifera* winegrapes, to identify these fungi to the species level, and to determine if they produce toxins in vitro. To the best of our knowledge, this study is the first report of *F. fujikuroi* isolated from winegrapes and the first report of a population of *F. fujikuroi* producing significant levels of fumonisins comparable to *F. verticillioides* and *F. proliferatum*.

## 2. Results

### 2.1. Fungal Isolates Collected from Winegrapes

A total of three black-spored *Aspergillus* isolates were recovered from the winegrape samples, each one from a different vineyard. None of these isolates had the morphological characteristics of the main OTA producer, *A. carbonarius*, and thus were not further investigated for mycotoxin production. In contrast, a total of 314 *Fusarium* isolates were recovered from the winegrape samples using dichloran rose bengal chloramphenicol (DRBC) and 2-benzoxazolinone (BOA) selective media. Based on sequencing of the translation elongation factor 1-α (TEF) region and BLASTN searches of three nucleotide databases for species identification, *F. fujikuroi* was the most abundant species recovered (*n* = 239, 76.1% of isolates), followed by *F. proliferatum* (*n* = 52, 16.6%), *F. incarnatum-equiseti* (*n* = 14, 4.5%), *F. oxysporum* (*n* = 7, 2.2%), *F. concentricum* (*n* = 1, <1%), and *F. solani* (*n* = 1; <1%). *F. fujikuroi* isolates were recovered from every vineyard (*n* = 8) and every grape variety (*n* = 10) sampled (Table 1), whereas *F. proliferatum* was recovered from fewer vineyards (*n* = 3) and grape varieties (*n* = 4), and only from the states of North Carolina and Alabama. The importance of collecting more than one isolate from a single sample replicate was demonstrated, as multiple species were found in distinct replicates.

To further support the determination that isolates were either *F. fujikuroi* or *F. proliferatum*, percent identity matrices were generated from ClustalW nucleotide alignments of winegrape isolates compared to multiple reference strains of each species. Identity of ≥97% was used to make species determinations, and *F. fujikuroi* was clearly distinguishable from *F. proliferatum* (Supplementary File). Also, due to the large number of isolates obtained from the winegrapes, only representative isolates from across vineyards, grape varieties, and sample replicates were deposited in the Agricultural Research Service (ARS) Culture Collection (NRRL), and the TEF sequences from these representatives were deposited in the National Center for Biotechnology Information (NCBI) GenBank (Table 2). Percent identity matrices of TEF alignments for those isolates not curated in NRRL and GenBank are presented in the Supplementary File as support of their species determination.

### 2.2. In Vitro Fumonisin Production Analysis

A representative group of *F. proliferatum* and *F. fujikuroi* winegrape isolates were further explored for their fumonisin producing capabilities in vitro using a rice-based assay (Table 2). Nearly all tested *F. proliferatum* isolates (20/22) produced fumonisins in the range of 1125–4653 ppm (µg/g) total fumonisins (FB1, FB2, and FB3). Average total fumonisins for *F. proliferatum* isolates was 1922 ppm. All *F. fujikuroi* isolates (50/50) produced fumonisins with an average total fumonisins of 1997 ppm (range of 1.09–3921 ppm). The average total fumonisins produced by the positive control, *F. verticillioides*, was 1675 ppm. No *F. oxysporum* isolates tested produced fumonisins in this study. There were no correlations with fumonisin production and state, vineyard, grape variety, or media isolation type (DRBC or BOA). Furthermore, isolates with the TEF SNP corresponding with non-production according to Suga et al. [26] did produce fumonisins in this study (Table 2).

## 3. Discussion

Whereas *F. proliferatum* is a well-known fumonisin producer, the general consensus in the *Fusarium* research community has been that *F. fujikuroi* produces little or no fumonisins. Surprisingly, all but two *F. fujikuroi* winegrape isolates tested with the rice assay produced significant levels of total fumonisins (greater than 400 ppm; either B1, B2, B3, or a combination). The levels of fumonisins produced by the *F. fujikuroi* winegrape isolates are comparable to the levels produced by the *F. proliferatum* winegrape isolates and to the *F. verticillioides* positive controls. This is the first report of a large population of *F. fujikuroi* isolates with the ability to produce high amounts of fumonisins.

Due to the rarity of a high fumonisin-producing *F. fujikuroi* population, further testing with verified *F. fujikuroi* and *F. proliferatum* isolates from the NRRL Culture Collection was conducted to determine if the rice assay method of in vitro fumonisin production, less common than the alternative cracked corn assay, was perhaps the cause of such production. One of these verified *F. fujikuroi* isolates, NRRL 31883, was isolated from a grape host in NY, USA. This one isolate, but none of the other NRRL *F. fujikuroi* reference strains we tested, produced high amounts of fumonisins similar to the southeastern winegrape isolates (Table 3). All other *F. fujikuroi* NRRL reference strains produced undetectable to low amounts of fumonisins (<5 ppm). The species identity of all NRRL isolates tested was verified through sequencing of the TEF region as described earlier. Therefore, the rice assay testing method was not the cause of the high fumonisin production by *F. fujikuroi* winegrape isolates, which represent a truly unique population for this species. Differential regulation of the fumonisin biosynthetic gene cluster within this population may be the basis for the enhanced production of the mycotoxin. The grape host or the associated vineyard pest management practices may create an environment conducive to activation of the biosynthetic genes, perhaps related to nitrogen availability, which was shown to affect expression of the gene cluster [31]. Further studies would be needed to address these possibilities. We do wish to note that our laboratory has conducted fumonisin testing of southeastern American wines, and to date no wine samples have contained fumonisins at a level which would pose a consumer health risk (unpublished data).

Comparing published results of fumonisin production by *Fusarium* isolates is difficult due to the great diversity amongst in vitro fumonisin testing assays. Methods include a variety of growth media (cracked corn, rice, liquid broth, agar plates), incubation periods, toxin extraction methods, and means of reporting the toxin levels (ppm as µg toxin per gram of corn, µg toxin per gram dry fungal mass, etc.). Therefore, a more accurate and comprehensive approach is to obtain standard strains from a well-curated repository, verify their identity through sequencing, and test these strains in-house alongside collected experimental isolates. To this end, 35 of the winegrape *Fusarium* isolates were deposited into the permanent ARS Culture Collection. The NRRL numbers of these winegrape isolates are listed in Table 2. These strains may be useful to other researchers for in-house comparison of fumonisin production and genetic studies related to better understanding the *F. fujikuroi* secondary metabolite profile and its regulation. Given the wide host range of *Fusarium* species, this newly discovered high fumonisin-producing population of *F. fujikuroi* in vineyards in the Southeast raises concern for fumonisin contamination in other crops as well. Closer attention should be paid to the mycotoxigenic potential of *F. fujikuroi* worldwide.

## 4. Materials and Methods

### 4.1. Collection of Winegrape Samples

Samples of red, *V. vinifera* winegrapes were obtained from eight commercial vineyards in the southeastern U.S. (AL, GA, NC) during harvest in 2013. This harvest represented a vintage with both late season rains and high disease pressure. Grape varieties sampled included Cabernet Sauvignon, Cabernet franc, Malbec, Merlot, Petit Verdot, Pinot noir, Sangiovese, Tannat, and Touriga Nacional. One additional vineyard, planted with the non-*vinifera* variety of Norton, was also sampled. Up to three different grape varieties were sampled per vineyard. The majority of grape varieties sampled in each vineyard consisted of six clusters in total, each cluster randomly selected from a different non-adjacent grapevine on an individual vineyard row, forming up to three sample replicates of two clusters each. In total, 56 sample replicates were collected and processed. All clusters were brought to the laboratory on ice in re-sealable plastic bags and prepared for further analysis within 12 h from being cut from the vine. Stems were removed from clusters and grapes were homogenized (Polytron PT 10-35, Brinkmann Instruments, Thermo Fisher Scientific, Waltham, MA, USA) into a must slurry. The grapes were not surface sterilized in order to recover greater mycoflora diversity and to allow recovery of ochratoxigenic *A. carbonarius* or other black-spored *Aspergillus* species that typically sporulate on the grape berry exterior [15]. A sterile glycerin (Columbus Chemical Industries, Inc., Columbus, OH, USA) solution was added to grape must to a final concentration of 4%, and the must solution was frozen at −80 °C until further analysis.

### 4.2. Fungal Isolation

Thawed grape must sample replicates (1–3 per variety per vineyard) were serially diluted in sterile reverse osmosis (RO) water with 0.10% Tween^®^ 20 (Fisher Scientific, Fair Lawn, NJ, USA). Undiluted and serially diluted must samples were plated in duplicate onto two types of selective media: DRBC agar [32] and BOA agar [33]. Negative controls for each media type were included on every day of plating. Following a 5–7 day incubation period of DRBC plates at 30 °C and a 7–10 day incubation period of BOA plates at 27 °C, all potential black-spored *Aspergillus* and cottony-white *Fusarium* colonies were transferred onto potato dextrose agar (PDA) and incubated at 27 °C or 30 °C for purification and further identification. As *Fusarium* spp. were abundant, a maximum of 10 *Fusarium* colonies were isolated from each sample replicate and media type combination. Pure cultures of either *Aspergillus* or *Fusarium* were single-spore isolated via the dilution plating method on PDA [34]. Isolates were stored in 15% glycerin/0.1% Tween^®^ 20 at −80 °C for further analysis.

### 4.3. DNA Extraction and Isolate Identification

Single-spored *Fusarium* isolates were grown on PDA for 2–4 days at 27 °C and genomic DNA was extracted using the previously described thermolysis procedure [35]. The primers EF-1 (5′-GTTAAGAGGCGCGGTGTCGGTGTG-3′) and EF-2 (5′-GGAAGTACCAGTGATCATGTT-3′) [36], along with 1 µL of fungal genomic DNA and Taq Hot Start Quick-Load^®^ 2X Master Mix with Standard Buffer (New England BioLabs, Inc., Ipswich, MA, USA) were used to PCR-amplify a portion of the TEF gene for species identity [37]. PCR conditions were as follows: 94 °C for 30 s, followed by 39 cycles of 94 °C for 10 s, 55 °C for 10 s, and 68 °C for 45 s, with a final extension of 68 °C for 5 min. Gene amplification was verified by gel electrophoresis, then PCR products were sent to GenScript USA, Inc. (Piscataway, NJ, USA) for purification and DNA sequencing. Sequencing primers used were the same as PCR primers. Sequences were edited with Sequencher^®^ version 5.4.1 (Gene Codes Corporation, Ann Arbor, MI, USA). The TEF sequences were compared with verified strains in the *Fusarium* MLST (CBS-KNAW Fungal Biodiversity Centre, Utrecht, The Netherlands) [38], *Fusarium*-ID [39], and NCBI-BLAST [40] databases for species identification. Sequences from winegrape isolates were aligned to *F. fujikuroi* and *F. proliferatum* reference sequences from the databases using ClustalW implemented in Geneious analysis software (version 8.1.8, 2015, Biomatters Limited, Auckland, New Zealand) [41]. Percent nucleotide identity matrices were generated from the alignments, and an identity of ≥97% to reference isolates was used to designate winegrape isolates as either *F. fujikuroi* or *F. proliferatum*. Detailed phylogenetic characterization of these isolates will be published separately.

### 4.4. In Vitro Fumonisin Production Analysis

Following species identification, a subset (*n* = 73) of the total recovered potentially mycotoxigenic *Fusarium* winegrape isolates (*n* = 291) was selected for in vitro fumonisin analysis. This representative subset included both *F. proliferatum* (*n* = 22) and *F. fujikuroi* (*n* = 51) isolates from different states, vineyards, grape varieties, sample replicates, and media isolation types. Additionally, to test the Japanese isolate hypothesis that a SNP indicates fumonisin production or lack thereof, representative isolates with both producing and non-producing SNP types were included [26]. Four *F. oxysporum* winegrape isolates were assayed as well, though fumonisin B production is extremely rare for this species. Isolates of *F. incarnatum*-*equiseti* and *F. concentricum*, also from winegrapes, were included as negative controls. Additionally, strains of *F. verticillioides*, *F. proliferatum*, and *F. fujikuroi* were obtained from the USDA-ARS Culture Collection (NRRL) for inclusion in the fumonisin analysis (Peoria, IL, USA) [42]. Fumonisin-producing *F. verticillioides* strains (NRRL 20956, NRRL 20960, NRRL 20984) were used as positive controls, and one winegrape *F. fujikuroi* isolate (SLB 6326) that produced fumonisins in the first assay was used as an internal assay control for all subsequent assays. Therefore, each experimental run included testing potential fumonisin-producing *Fusarium* isolates from winegrapes and/or verified strains from the NRRL Culture Collection, a winegrape isolate negative control, a sterile water negative control, a *F. verticillioides* positive control, and an internal assay control. All isolates and strains analyzed, including controls, were tested in triplicate in each experiment. Assays were repeated on different days, and every winegrape isolate and verified strain was tested for fumonisin production in at least two independent experiments.

For fumonisin analysis, fungal isolates were grown on sterile rice using an assay modified from [27]. For each assay run, approximately 10 µL of a high-inoculum spore suspension from frozen single-spored stock cultures (−80 °C, in 15% glycerin/0.1% Tween^®^ 20) was used as inoculum for 50 mL of sterile potato dextrose broth (PDB) in a 125 mL Erlenmeyer shaking flask. Culture flasks were incubated for 3 days (±1 h) at 27 °C with shaking at 200 rpm in darkness. To a 20 mL glass scintillation vial, 3 g (±0.004 g) of rice (enriched long grain rice, Kroger, Athens, GA, USA) and 3 mL of reverse osmosis water were added. Vials were topped with foam plugs, autoclaved (121 °C, 30 min), and cooled to room temperature just prior to use. In triplicate, 250 µL of high inoculum spore suspension (~10^7^ spores per mL) from incubated PDB flasks (or sterile water control) were added to each rice vial replicate. Inoculated rice vials were incubated for 7 days (±1 h) at 27 °C in darkness.

**Figure 1:**
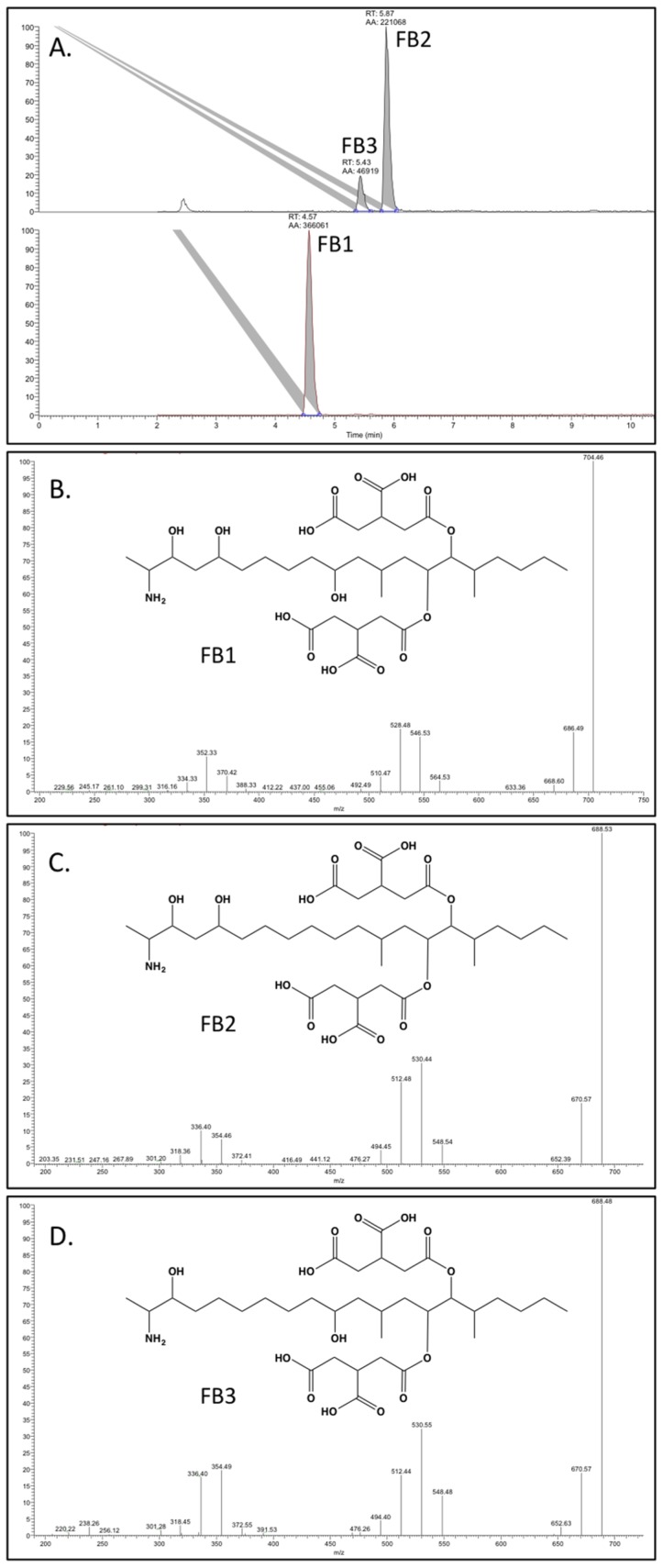
Liquid chromatography-tandem mass spectrometry (LC-MS/MS) analysis of fumonisins produced by *F. fujikuroi* winegrape isolate SLB 6214. (**A**) Chromatogram showing signature peaks for FB1, FB2, and FB3 with retention times of 4.57, 5.87, 5.43 min, respectively. The structure and daughter ions (*m/z*) for (**B**) FB1; (**C**) FB2; and (**D**) FB3.

Following the incubation period on rice, 10 mL of a 1:1 acetonitrile (HPLC grade, Fisher, Fair Lawn, NJ, USA): distilled water extraction solution containing 5% formic acid (~98%, Sigma-Aldrich, St. Louis, MO, USA) was added to each vial. Samples were vigorously shaken and frozen to −20 °C for at least 10 h. Prior to LC-MS/MS analysis for fumonisins, rice cultures were broken apart and vials were rocked for 3 h (The Belly Dancer^®^, speed 6.5, Stovall Life Science, Inc., Greensboro, NC, USA) to extract any fumonisins. The resulting extraction solution was centrifuge-filtered (costar^®^ Spin-X HPLC Micro Centrifuge Filter, 0.45 µm, nylon, Corning Inc., Corning, NY, USA), diluted (up to 10,000 fold), and analyzed via LC-MS/MS (Thermo LTQ XL). All analyses were multiple reaction monitoring (MRM) performed in positive ion mode, and the instrument was tuned using FB1 (PROMEC, Tygerberg, South Africa). An Imtakt Cadenza CW-C18 column (150 × 2 mm, 3 µm; Kyoto, Japan), maintained at 30 °C, was used for separations. Solvent A was distilled water with 3% acetonitrile plus 0.1% formic acid, and solvent B was acetonitrile with 3% distilled water plus 0.1% formic acid. The solvent program began at 30:70 A:B, and increased linearly to 100% B over 9 min, held at 100% B for 2 min, and then returned to starting conditions. Samples were run along with a blank control and standard solutions of fumonisins B1, B2, and B3 (1 ppb, 10 ppb, and 100 ppb in 30:70 acetonitrile:distilled water plus 0.1% formic acid; PROMEC, Tygerberg, South Africa). Analytes were identified by retention time, as compared to standards, and fragmentation patterns (Figure 1). Levels of fumonisins were quantified by external calibration of peak areas (the MS/MS total ion current) with standards, and a new calibration curve (limit of quantification (LOQ) = 0.045 ppb and limit of detection (LOD) = 0.013 ppb) was run with each sample set. Fumonisin amounts were reported as µg toxin per gram of dried rice (ppm).

## Figures and Tables

**Table 1:** A high frequency of *Fusarium fujikuroi* isolates were recovered from southeastern winegrapes.

Vineyard	State	# of Sample Replicates ^a^	Total # of *Fusarium* Isolates	# of *Fusarium fujikuroi* Isolates	% *Fusarium fujikuroi*
01	GA	9	19	19	100%
02	GA	5	9	8	88.9%
05	NC	9	99	89	89.9%
09	NC	10	72	44	61.1%
12	GA	2	38	38	100%
16 ^b^	AL	3	43	7	16.3%
18	GA	9	28	28	100%
20	GA	9	6	6	100%

^a^ One sample replicate is equivalent to two grape clusters. From each vineyard, three sample replicates were collected (where possible) from one to three different grape varieties; ^b^ Vineyard 16 grape samples (the only non-*vinifera* variety, Norton) were collected approximately seven days post-harvest. All other samples were collected at commercial vineyards on the day of or a few days prior to harvest time.

**Table 2:** In vitro fumonisin production by winegrape *Fusarium* isolates and *F. verticillioides* maize isolates.

Species Based on TEF Seq.	State (USA) or Country	Vineyard-Variety- Replicate ^a^	Isolate No. Assigned	NRRL No. ^b^	GenBank TEF Accession ^b^	SNP Type ^c^	FB1 (ppm) ^d^	FB2 (ppm) ^d^	FB3 (ppm) ^d^	Total FB (ppm)	Fumonisin Production ^e^
*F. concentricum*	NC	05-CF-1	SLB 6283	66449	KX711881	-	ND	ND	ND	ND	ND
*F. fujikuroi*	GA	01-SA-2	SLB 6163	-	-	Y	975	848	150	1973	HIGH
*F. fujikuroi*	GA	01-SA-2	SLB 6254	66445	KX656201	Y	968	769	114	1850	HIGH
*F. fujikuroi*	GA	01-SA-2	SLB 6184	-	-	Y	2403	1160	329	3891	HIGH
*F. fujikuroi*	GA	01-SA-3	SLB 6185	66431	KX656193	Y	1421	1194	301	2916	HIGH
*F. fujikuroi*	GA	01-TN-2	SLB 6258	-	-	N	616	412	16	1043	MEDIUM
*F. fujikuroi*	GA	01-TN-3	SLB 6262	66446	KX656202	Y	1706	327	139	2172	HIGH
*F. fujikuroi*	GA	01-TN-3	SLB 6270	-	-	Y	2336	524	265	3125	HIGH
*F. fujikuroi*	GA	02-TA-2	SLB 6309	66452	KX656206	Y	1037	926	165	2128	HIGH
*F. fujikuroi*	GA	02-TN-2	SLB 6128	66427	KX656189	Y	947	626	114	1687	HIGH
*F. fujikuroi*	NC	05-CS-1	SLB 6164	66428	KX656190	Y	647	446	51	1144	MEDIUM
*F. fujikuroi*	NC	05-CS-1	SLB 6172	-	-	Y	720	566	84	1370	MEDIUM
*F. fujikuroi*	NC	05-CS-2	SLB 6113	66425	KX656187	Y	1482	1076	121	2678	HIGH
*F. fujikuroi*	NC	05-CS-2	SLB 6174	-	-	Y	1258	1116	261	2635	HIGH
*F. fujikuroi*	NC	05-CS-3	SLB 6092	-	-	Y	1661	730	179	2570	HIGH
*F. fujikuroi*	NC	05-CS-3	SLB 6118	66426	KX656188	Y	1663	750	185	2598	HIGH
*F. fujikuroi*	NC	05-CF-1	SLB 6282	66448	KX656204	Y	0.85	0.05	0.19	1.09	LOW
*F. fujikuroi*	NC	05-CF-2	SLB 6281	66447	KX656203	Y	9	1640	1	1649	HIGH-FB2
*F. fujikuroi*	NC	05-ME-1	SLB 6204	-	-	Y	1282	700	145	2126	HIGH
*F. fujikuroi*	NC	05-ME-1	SLB 6214	66439	KX656197	Y	1275	711	149	2134	HIGH
*F. fujikuroi*	NC	05-ME-2	SLB 6224	66441	KX656198	Y	1147	917	207	2271	HIGH
*F. fujikuroi*	NC	05-ME-2	SLB 6236	66444	KX656200	Y	2	1728	ND	1730	HIGH-FB2
*F. fujikuroi*	NC	05-ME-3	SLB 6234	66443	KX656199	N	1961	455	49	2465	HIGH
*F. fujikuroi*	NC	05-ME-3	SLB 6239	-	-	N	1806	474	52	2331	HIGH
*F. fujikuroi*	NC	05-ME-3	SLB 6240	-	-	Y	1153	1022	282	2456	HIGH
*F. fujikuroi*	NC	09-CS-1	SLB 6020	-	-	Y	976	937	230	2142	HIGH
*F. fujikuroi*	NC	09-CS-1	SLB 6012	66415	KX656183	Y	1627	1217	385	3229	HIGH
*F. fujikuroi*	NC	09-CS-1	SLB 6317	-	-	Y	1202	572	73	1846	HIGH
*F. fujikuroi*	NC	09-CS-3	SLB 6300	-	-	N	642	172	21	835	MEDIUM
*F. fujikuroi*	NC	09-CS-3	SLB 6326	66453	KX656207	N	826	209	13	1049	MEDIUM
*F. fujikuroi*	NC	09-PN-1	SLB 6023	66418	KX656184	Y	1180	248	156	1584	HIGH
*F. fujikuroi*	NC	09-PN-1	SLB 6005	-	-	Y	1055	420	164	1638	HIGH
*F. fujikuroi*	GA	12-CS-1	SLB 6190	-	-	Y	1120	1028	283	2430	HIGH
*F. fujikuroi*	GA	12-CS-1	SLB 6196	-	-	Y	1046	958	248	2252	HIGH
*F. fujikuroi*	GA	12-CS-1	SLB 6177	-	-	Y	906	817	137	1861	HIGH
*F. fujikuroi*	GA	12-CS-2	SLB 6140	-	-	Y	1815	830	175	2819	HIGH
*F. fujikuroi*	GA	12-CS-2	SLB 6180	66429	KX656191	Y	1520	1821	580	3921	HIGH
*F. fujikuroi*	AL	16-NO-1	SLB 6051	66421	KX656185	Y	41	46	5	92	LOW
*F. fujikuroi*	AL	16-NO-3	SLB 6064	-	-	N	714	351	11	1076	MEDIUM
*F. fujikuroi*	AL	16-NO-3	SLB 6069	66423	KX656186	Y	355	36	11	401	LOW
*F. fujikuroi*	GA	18-MA-3	SLB 6257	-	-	Y	1032	966	233	2231	HIGH
*F. fujikuroi*	GA	18-CF-1	SLB 6072	-	-	Y	1011	952	239	2201	HIGH
*F. fujikuroi*	GA	18-CF-1	SLB 6109	-	-	Y	1028	1029	221	2277	HIGH
*F. fujikuroi*	GA	18-PV-1	SLB 6278	-	-	N	840	390	29	1259	MEDIUM
*F. fujikuroi*	GA	18-PV-1	SLB 6289	66450	KX656205	N	574	268	12	853	MEDIUM
*F. fujikuroi*	GA	18-PV-1	SLB 6284	-	-	N	720	347	25	1092	MEDIUM
*F. fujikuroi*	GA	20-MA-2	SLB 6203	66438	KX656196	Y	1856	777	165	2798	HIGH
*F. fujikuroi*	GA	20-MA-3	SLB 6183	66430	KX656192	Y	1348	899	179	2425	HIGH
*F. fujikuroi*	GA	20-TA-1	SLB 6199	66432	KX656194	Y	1561	692	106	2359	HIGH
*F. fujikuroi*	GA	20-TA-2	SLB 6201	66437	KX656195	Y	1502	550	143	2195	HIGH
*F. fujikuroi*	GA	20-TA-2	SLB 6200	-	-	Y	1402	509	123	2033	HIGH
*F. oxysporum*	GA	02-TN-1	SLB 6150	-	-	n/a	ND	ND	ND	ND	ND
*F. oxysporum*	NC	09-PN-1	SLB 6007	-	-	n/a	ND	ND	ND	ND	ND
*F. oxysporum*	NC	09-ME-2	SLB 6265	-	-	n/a	ND	ND	ND	ND	ND
*F. oxysporum*	AL	16-NO-3	SLB 6063	-	-	n/a	ND	ND	ND	ND	ND
*F. proliferatum*	NC	05-CS-2	SLB 6099	-	-	n/a	ND	ND	1723	1723	HIGH-FB3
*F. proliferatum*	NC	05-CS-2	SLB 6100	66424	KX656219	n/a	ND	300 ^f^	3118	3418	HIGH-FB3
*F. proliferatum*	NC	05-CS-2	SLB 6115	-	-	n/a	3503	703	447	4653	HIGH
*F. proliferatum*	NC	05-ME-2	SLB 6228	66442	KX656220	n/a	1491	132	313	1936	HIGH
*F. proliferatum*	NC	05-ME-2	SLB 6237	-	-	n/a	1681	150	326	2156	HIGH
*F. proliferatum*	NC	09-CS-1	SLB 6016	66417	KX656215	n/a	ND	ND	ND	ND	ND
*F. proliferatum*	NC	09-CS-2	SLB 6022	-	-	n/a	1012	136	95	1243	MEDIUM
*F. proliferatum*	NC	09-CS-2	SLB 6013	66416	KX656214	n/a	1094	146	110	1349	MEDIUM
*F. proliferatum*	NC	09-CS-2	SLB 6014	-	-	n/a	985	136	101	1222	MEDIUM
*F. proliferatum*	NC	09-CS-2	SLB 6322	-	-	n/a	881	328	86	1295	MEDIUM
*F. proliferatum*	NC	09-CS-2	SLB 6298	66451	KX656221	n/a	771	294	61	1125	MEDIUM
*F. proliferatum*	NC	09-CS-2	SLB 6327	-	-	n/a	971	368	98	1436	MEDIUM
*F. proliferatum*	NC	09-CS-2	SLB 6332	-	-	n/a	937	352	91	1380	MEDIUM
*F. proliferatum*	NC	09-PN-1	SLB 6028	-	-	n/a	1388	479	108	1974	HIGH
*F. proliferatum*	NC	09-PN-1	SLB 6009	-	-	n/a	1488	497	110	2095	HIGH
*F. proliferatum*	AL	16-NO-1	SLB 6046	-	-	n/a	1676	739	258	2673	HIGH
*F. proliferatum*	AL	16-NO-1	SLB 6042	66419	KX656216	n/a	2262	898	369	3528	HIGH
*F. proliferatum*	AL	16-NO-1	SLB 6045	66420	KX656217	n/a	ND	ND	ND	ND	ND
*F. proliferatum*	AL	16-NO-2	SLB 6038	-	-	n/a	1886	244	138	2268	HIGH
*F. proliferatum*	AL	16-NO-2	SLB 6056	66422	KX656218	n/a	1642	242	90	1974	HIGH
*F. proliferatum*	AL	16-NO-2	SLB 6061	-	-	n/a	2095	274	182	2550	HIGH
*F. proliferatum*	AL	16-NO-2	SLB 6052	-	-	n/a	1880	270	127	2276	HIGH
*F. verticillioides*	CA	-	+ control	20956	n/a	n/a	663	104	26	793	MEDIUM
*F. verticillioides*	S. Africa	-	+ control	20960	n/a	n/a	956	499	10	1464	MEDIUM
*F. verticillioides*	IN	-	+ control	20984	n/a	n/a	1695	933	141	2769	HIGH

^a^ Identification system used in study. Two-digit codes were assigned to vineyards. Grape varieties are coded as follows: CS = Cabernet Sauvignon, CF = Cabernet Franc, ME = Merlot, MA = Malbec, NO = Norton, TA = Tannat, SA = Sangiovese, TN = Touriga Nacional, PN = Pinot Noir, PV = Petit Verdot. The last digit refers to the replicate from which the isolate was obtained; ^b^ A subset of representative isolates from this study were deposited in the Agricultural Research Service (ARS) Culture Collection (NRRL). The translation elongation factor (TEF) sequences of these curated isolates were deposited in the National Center for Biotechnology Information (NCBI) GenBank. Other winegrape isolates, indicated by a dash, were sequenced to confirm species identity, but sequences were not deposited in GenBank since the isolates were not curated in NRRL. n/a = not applicable to *F. verticillioides* positive controls; ^c^ Y = SNP (single nucleotide polymorphism in TEF region) identified by Suga et al. [26] to be a fumonisin producer. N = SNP identified to be a non-producer. n/a = the SNP applies to *F. fujikuroi* and is not applicable to other species; ^d^ Represents the average of a minimum of two independent experiments, each with three technical replicates. Unit = ppm = µg toxin per gram of dried rice. ND = none detected, limit of detection (LOD) = 0.013 ppb, and limit of quantification (LOQ) = 0.045 ppb; ^e^ ND = none detected, LOW = <500 ppm, MEDIUM = 500–1500 ppm, HIGH = >1500 ppm; ^f^ SLB 6100 is the only isolate tested which had discrepancy in production of FB2 between assays. In three of the four assay replicates (each tested in triplicate), this isolate produced no detectable FB2, but in one assay replicate it produced 1200 ppm average FB2.

**Table 3:** In vitro fumonisin production by NRRL verified *Fusarium* strains.

Species (TEF)	Source Host	Geographic Origin	NRRL ID ^a^	GenBank TEF Accession	FB1 (ppm) ^b^	FB2 (ppm) ^b^	FB3 (ppm) ^b^	Total FB (ppm)	Fumonisin Production ^c^
*F. fujikuroi*	sugarcane	Taiwan	5538	HF679028	3	0.6	ND	3.6	LOW
*F. fujikuroi*	rice	Taiwan	13566	AF160279	ND	ND	ND	ND	ND
*F. fujikuroi*	rice	Taiwan	13998	AB725605	ND	ND	ND	ND	ND
*F. fujikuroi*	rice	Taiwan	22012	JN092354	3.5	ND	ND	3.5	LOW
*F. fujikuroi*	soil	China	31857	KX656178	ND	ND	ND	ND	ND
*F. fujikuroi*	rice	GA, USA	31862	KX656179	3	ND	ND	3	LOW
*F. fujikuroi*	C tester strain	USA	31879	KX656180	ND	ND	ND	ND	ND
*F. fujikuroi*	grape	NY, USA	31883	KX656181	1334	1657	370	3360	HIGH
*F. fujikuroi*	rice	Nepal	31886	KX656182	2	ND	ND	2	LOW
*F. proliferatum*	flower	Germany	22944	AF160280	412	104	129	644	MEDIUM
*F. proliferatum*	soil	China	31860	KX656208	2770	650	190	3610	HIGH
*F. proliferatum*	fishtail palm	Australia	31865	KX656209	1225	136	74	1435	MEDIUM
*F. proliferatum*	peanut	GA, USA	31866	KX656210	1825	735	91	2650	HIGH
*F. proliferatum*	date palm	Iraq	31867	KX656211	1089	498	134	1721	HIGH
*F. proliferatum*	mango	Malaysia	31871	KX656212	945	621	87	1652	HIGH
*F. proliferatum*	Bakanae rice	Thailand	31876	none	1448	165	148	1761	HIGH
*F. proliferatum*	rice	MS, USA	31915	KX656213	3589	653	319	4561	HIGH
*F. verticillioides*	rice	China	22001	AY337449	2680	752	132	3563	HIGH
*F. verticillioides*	maize	Germany	22172	AF160262	2008	925	118	3051	HIGH

^a^ NRRL = USDA-ARS Culture Collection (more strain info. available at http://nrrl.ncaur.usda.gov/); ^b^ Represents the average of a minimum of two independent experiments, each with three technical replicates. Unit = ppm = µg toxin per gram of dried rice. ND = none detected, LOD = 0.013 ppb, LOQ = 0.045 ppb; ^c^ ND = none detected, LOW = <500 ppm, MEDIUM = 500–1500 ppm, HIGH = >1500 ppm.

## Supplementary Materials

The following are available online at www.mdpi.com/2072-6651/8/9/254/s1.
